# Incidence, Prevalence, and Risk Factors of Dog Bites in Pediatric Populations: Protocol for a Systematic Review

**DOI:** 10.2196/103027

**Published:** 2026-08-27

**Authors:** Nathaniel LaBarre, Michael Karo, Vidhani Goel, Roberto Sagaribay, Vinnesa Narvaez, Ami Shah, Aditi Singh, Fatehali Peera, Kavita Batra

**Affiliations:** 1 Department of Medical Education, Kirk Kerkorian School of Medicine at UNLV, University of Nevada, Las Vegas, 1701 W. Charselton, Las Vegas, NV, 89102, United States, 1 702-823-3751; 2 Office of Research, Kirk Kerkorian School of Medicine at UNLV, University of Nevada, Las Vegas, Las Vegas, NV, United States; 3 Pediatric Emergency Medicine, Kirk Kerkorian School of Medicine at UNLV, University of Nevada, Las Vegas, Las Vegas, NV, United States; 4 Pediatric Emergency Medicine, University Medical Center of Southern Nevada, Las Vegas, NV, United States; 5 Department of Internal Medicine, Kirk Kerkorian School of Medicine at UNLV, University of Nevada, Las Vegas, Las Vegas, NV, United States

**Keywords:** dog bites, pediatric injuries, systematic review, epidemiology, risk factors, public health, bite prevention, emergency medicine, child safety, incidence and prevalence

## Abstract

**Background:**

Dog bites represent a significant global public health concern, particularly among children and adolescents. Approximately 330,000 emergency department visits occur annually in the United States alone due to dog-bite injuries, with pediatric populations bearing a disproportionate burden. Despite this, existing systematic reviews remain fragmented, focusing on isolated aspects such as breed-specific risk, educational interventions, or legislative measures, without comprehensively addressing the global incidence, prevalence, and multifactorial risk profile in pediatric cohorts.

**Objective:**

This protocol describes a planned systematic review aimed at synthesizing international evidence on the incidence and prevalence of dog bites in pediatric populations (aged ≤18 y), with concurrent analysis of demographic, behavioral, and environmental risk and protective factors. Secondary objectives are to characterize the clinical severity, psychological sequelae, and health economic consequences of pediatric dog-bite injury as reported in the eligible literature.

**Methods:**

This protocol is reported in accordance with the PRISMA-P (Preferred Reporting Items for Systematic Review and Meta-Analysis Protocols) 2015 statement. A comprehensive search of PubMed, Embase, Web of Science, Scopus, CINAHL, and the World Health Organization Global Index Medicus, supplemented by forward and backward citation tracking and gray literature sources, will be conducted with no date restrictions and limited to English-language observational studies (cross-sectional, case-control, and cohort) and population-based surveys. The PECOS (population, exposure, comparator, outcome, and study design) framework will guide inclusion and exclusion criteria. Two independent reviewers will screen records and extract data using a standardized codebook, with discrepancies resolved by consensus or by a third reviewer. Study quality will be assessed using validated tools appropriate for each study design. Findings will be synthesized narratively with descriptive statistics. When studies are sufficiently homogeneous, prevalence and incidence will be pooled using random-effects meta-analysis of Freeman-Tukey double arcsine–transformed proportions, with prediction intervals reported alongside pooled estimates. The certainty of evidence for each outcome will be rated using the GRADE (Grading of Recommendations Assessment, Development, and Evaluation) approach.

**Results:**

The systematic database search across PubMed, Embase, and Web of Science was conducted on August 1, 2025. At the time of manuscript submission, study screening and data extraction are ongoing. The anticipated completion of data extraction and evidence synthesis is in December 2026, with submission of the completed systematic review for publication expected thereafter. As this manuscript describes the review protocol, no study findings are available at this time.

**Conclusions:**

By aggregating data across diverse populations and settings, this systematic review will deliver the most comprehensive global assessment of pediatric dog-bite epidemiology to date, stratified by age group, geographic region, sex, and socioeconomic context, with direct implications for reducing the burden of these preventable injuries worldwide.

## Introduction

Dog-bite injuries represent a significant global public health burden with substantial geographic and demographic variation. In the United States, over 4.5 million incidents occur annually, with nearly 800,000 requiring medical attention [1,2], while reported incidence rates vary considerably across other regions, ranging from 15.8 per 100,000 in Greece [3] to 87.51 per 100,000 in Uruguay [4]. Several countries demonstrate concerning trends, with New Zealand reporting a 1.75% annual increase in dog-related injury claims [5], though mortality rates remain stable in some high-income settings [6]. Pediatric populations are disproportionately affected, with children under 10 years of age experiencing higher hospitalization rates and more severe injuries, particularly to the head and neck region [2,5,7]. Notable health inequities exist, including higher rates among Indigenous populations and those in areas of greater socioeconomic deprivation [5]. Despite this evidence of substantial burden, comprehensive global surveillance data remain limited, with significant gaps in understanding the full epidemiological picture, particularly regarding underreporting, economic costs, and systematic differences between high-income and low-income countries.

Multiple studies have identified risk factors for dog-bite injuries. Living with a dog, male sex, and low socioeconomic status have been linked to higher bite risk [8,9]. Children are uniquely vulnerable to dog bites due to a complex interplay of developmental, physical, and behavioral factors. Developmentally, children lack the cognitive capacity to recognize and interpret warning signs from dogs, often misinterpreting aggressive signals such as baring teeth as happy or smiling [10,11]. Additionally, their smaller stature places them at particular risk for head and neck injuries, as dogs’ mouths are at the same level as children’s faces [12-14]. This facial height vulnerability is compounded by children’s impulsive behavior and tendency toward risk-taking, especially in younger children who may approach dogs unpredictably without considering the consequences [11]. Inadequate supervision is another critical factor, with most dog bites occurring when young children are left alone with dogs without adult oversight [11]. Paradoxically, children are most often bitten by family dogs or dogs they know well, creating a false sense of security that can lead to inappropriate interactions and reduced vigilance from both children and caregivers [11,14]. This combination of developmental limitations, physical vulnerability, behavioral impulsivity, supervision gaps, and misplaced trust creates a uniquely dangerous situation for children around dogs.

Dog bites in children may also result in devastating physical and psychological consequences that extend far beyond the initial injury. Physically, infections occur in 28% of facial bite cases, with children under 5 years being 3 times more likely to develop infections [15]. The severity of injuries may necessitate extensive reconstructive surgery, with 8% to 16% of cases requiring operative intervention [16,17]. These procedures range from simple laceration repairs to complex reconstructions involving nerve and vessel repair, fracture reductions, and in severe cases, tissue expanders, and skin grafts [16]. The cosmetic outcomes can be particularly devastating since 62% to 65% of pediatric dog bites affect the highly visible head and face region, with the cheeks and lips being the most frequently injured structures [16,17], often resulting in permanent scarring and disfigurement.

The psychological harm is equally profound and often underestimated. Posttraumatic stress disorder (PTSD) emerges as the most common psychological consequence, with studies showing that 55% of children develop partial or complete PTSD within 1 month of a dog bite [14]. Children experience traumatic flashbacks, recurrent nightmares, generalized anxiety, hypervigilance, and specific phobias such as cynophobia [14]. Over 70% of children demonstrate new, concerning behaviors following a bite, including increased fearfulness, social withdrawal, and sleep disturbances [18]. These psychological sequelae can persist for years, significantly impacting social and emotional development, with studies documenting personality changes characterized by excessive caution and constricted thinking, lasting up to 4 years [14]. The long-term quality-of-life implications are substantial, as untreated traumatic experiences can lead to chronic psychological issues, functional disabilities, and additional psychiatric conditions [18]. Despite this evidence, most pediatric trauma centers (64.3%) lack standardized protocols to address these psychological consequences [18], highlighting a critical gap in comprehensive care for these injuries.

Existing systematic reviews on dog bites have primarily concentrated on discrete aspects of the problem, such as educational interventions for children, breed-specific injury risk, desexing practices, legislative strategies, or postbite treatment and infection prevention. While these efforts provide important contributions, several persistent gaps limit the evidence base (Table 1). Reviews of educational interventions consistently report improvements in knowledge and observed behavior but do not examine whether these changes translate into reductions in dog-bite incidence [19,20]. Other reviews, such as those evaluating desexing or legislative measures, are hindered by small sample sizes, high heterogeneity, and the inability to establish causal inferences, thereby limiting their applicability [21,22]. Moreover, many reviews are restricted to specific regions or populations, reducing the generalizability of findings. Additional reviews have focused narrowly on outcomes such as mortality, injury severity, or breed involvement, without synthesizing global incidence and prevalence data [23-27]. Collectively, these limitations underscore a fragmented literature that lacks a comprehensive understanding of the global burden of dog bites.

The proposed systematic review seeks to address these critical gaps by synthesizing international evidence on the incidence and prevalence of dog bites across diverse populations and settings, with a particular focus on the pediatric population, defined as individuals aged 18 years or younger. By collating and comparing data across geographic regions, this review will provide a more comprehensive estimate of the burden of dog bites and highlight demographic, environmental, and contextual risk factors that contribute to injury patterns. In doing so, it will extend beyond the limitations of prior reviews, inform prevention strategies, and establish an evidence base to guide policy and public health interventions aimed at reducing the global impact of dog bites.

**Table 1.** Gap analysis of existing systematic reviews on dog-bite injuries. A summary of previously published systematic reviews and meta-analyses of dog-bite injuries in humans and, where applicable, pediatric populations, detailing each review’s study design, the time frame of the included literature, key findings, methodological limitations, and the specific evidence gap identified^a^.

Author, year	Study design	Key findings	Limitations	Identified gap
Duperrex et al [19], 2009	Systematic review (3 studies)	Educational interventions improved children’s knowledge, attitudes, and behavior toward dogs.	Excluded adolescents; small sample size; no bite reduction data.	Limited demographics; lacked bite incidence outcome measures.
Shen et al [20], 2017	Systematic review and meta-analysis (all years to 2014)	Cognitive or behavioral programs improved knowledge and dog-interaction behavior.	Poor evidence quality; limited attention to policy or legal factors.	Focused only on behavioral interventions; no evaluation of bite outcomes.
D'Onise et al [21], 2017	Systematic review (Ovid 1946 to Dec 2015)	Nondesexed dogs had higher bite risk than desexed dogs.	Small, heterogeneous studies; causal inference not possible.	Focused solely on desexing; not specific to pediatric populations.
Duncan-Sutherland et al [5], 2022	Systematic review (1960 to 2021)	Some evidence that dog control legislation reduces bite rates.	High heterogeneity; many low-quality studies.	Few prevention studies focused on children beyond educational settings.
Barrios et al [24], 2021	Systematic review and meta-analysis (2013 to 2017)	Fatal bites mostly affected older males (50‐64 y) in public settings.	Few reports and missing breed data limited conclusions.	Focused on fatalities; limited pediatric relevance.
Shakerian and Sadraei [23], 2023	Systematic review and meta-analysis (2016 to 2021)	Classified populations at highest risk of animal bites in Iran.	High heterogeneity; limited authorship.	Focused on all animal bites in Iran; excluded global or pediatric-specific trends.
Bailey et al [25], 2020	Systematic review (1971 to 2018)	German Shepherds and Pit Bull–type breeds caused most severe bites.	Inconsistent breed reporting methods.	Examined only breed factors; excluded human or environmental variables.
Yoon et al [26], 2025	Systematic review (2013 to 2024)	Interventions shortened recovery; dog-safety videos improved children’s caution.	Few high-quality RCTs^b^; limited geographic diversity.	Focused on RCTs; limited to non-US, nonpediatric samples.
Patterson et al [27], 2022	Systematic review (1980 to 2020)	Children <9 years had highest injury burden; <6 years faced greater facial and head injury risk.	Unregistered PRISMA^c^; few education or psychosocial studies; limited geographic scope.	Not generalizable; lacked data on triggering events or broader risk factors.

a The reviews span multiple countries and were published between 2009 and 2025.

b RCT: randomized controlled trial

c PRISMA: Preferred Reporting Items for Systematic Reviews and Meta-Analyses.

## Methods

### Ethical Considerations

As this study does not involve direct interaction with human participants, an institutional ethical review is not required. However, to adhere to rigorous methodology, the protocol of this systematic review has been prospectively registered in PROSPERO, an international database of prospectively registered systematic reviews, which provides each protocol with a unique, permanent registration number that prevents duplication, thereby reducing reporting bias.

### Protocol Reporting, Registration, and Amendments

**Figure 1.**
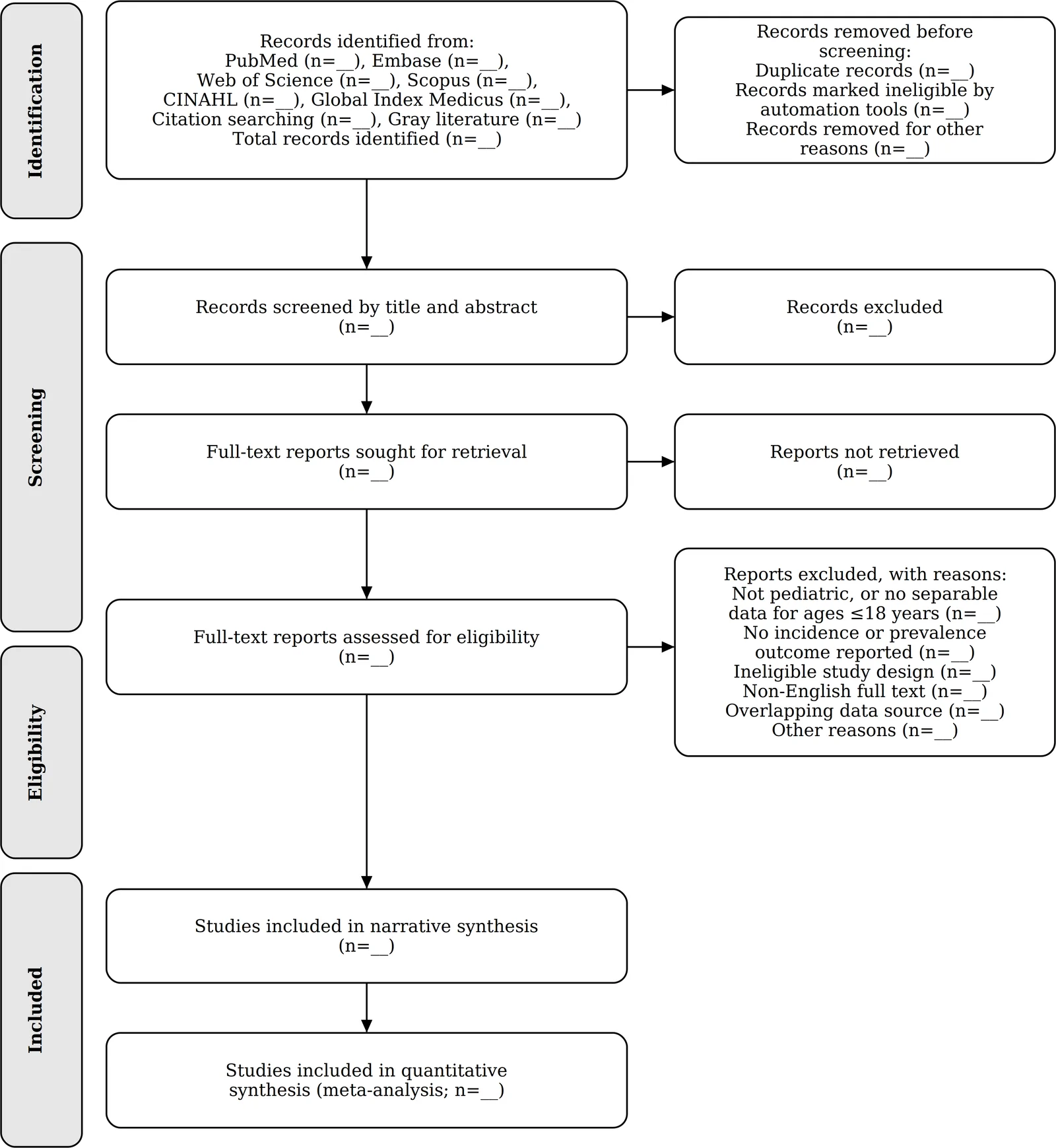
PRISMA (Preferred Reporting Items for Systematic Reviews and Meta-Analyses) 2020 flow diagram of study selection. Flow of records for a systematic review of the global incidence, prevalence, and risk factors of dog-bite injuries in pediatric populations (aged 18 years or younger), identified from PubMed, Embase, Web of Science, Scopus, CINAHL, Global Index Medicus, citation searching, and gray literature (database search conducted on August 1, 2025; no date restrictions; and English-language observational studies).

This protocol is reported in accordance with the PRISMA-P (Preferred Reporting Items for Systematic Review and Meta-Analysis Protocols) 2015 statement and its explanation and elaboration document [28,29]; the completed 17-item PRISMA-P checklist, with the corresponding manuscript page for each item, is provided as Checklist 1. PRISMA-P was selected in preference to the PRISMA (Preferred Reporting Items for Systematic Reviews and Meta-Analyses) 2020 statement because it is intended for the reporting of completed reviews rather than protocols. The completed systematic review will additionally be reported in accordance with the PRISMA 2020 statement [30], and the flow of records through the review will be presented using the PRISMA 2020 flow diagram (Figure 1).

Two substantive differences exist between the registered PROSPERO record (CRD420251129292) and the present protocol, both of which were introduced during peer review of this manuscript. First, the registered record specifies the incidence and prevalence of pediatric dog-bite injury as the review outcomes together with associated risk factors, whereas this protocol additionally specifies clinical severity, psychological sequelae, and health economic consequences as explicitly labeled secondary outcomes, so that the outcomes discussed in the *Introduction* section are represented consistently in the eligibility criteria, the data extraction form, and the analysis plan. Second, the registered record lists PubMed, Embase, and Web of Science as information sources, whereas this protocol expands the search to include Scopus, CINAHL, the World Health Organization Global Index Medicus, and gray literature sources in order to improve coverage in regions where dog-bite research is underindexed in the 3 original databases. Both amendments have been submitted to PROSPERO as formal updates to the registered record. Any further amendments to this protocol will be recorded in the PROSPERO register and reported in the final review with the date of the change, the rationale, and a description of what was changed, so that deviations from the planned methods can be distinguished from post hoc decisions.

### Review Question

Our review question is as follows: “Among the pediatric population, defined as individuals aged 18 years or younger worldwide, what are the prevalence and incidence of dog bites, and which demographic, behavioral, and environmental factors are associated with their occurrence, based on evidence from observational studies?”

### Inclusion and Exclusion Criteria

The PECOS (population, exposure, comparator, outcome, and study design) framework was used to formulate eligibility criteria for this systematic review and meta-analysis. Cross-sectional studies, case-control studies, retrospective and prospective cohort studies, and population-based surveys will be eligible for inclusion. Studies published in languages other than English, case reports, editorials, commentaries, posters, abstract-only studies, conference proceedings, animal studies, and review articles will be excluded. Studies that do not report quantitative data on the prevalence or incidence of dog bites, or that do not assess demographic, behavioral, or environmental predictors, will also be excluded. To be eligible, a study must report at least one primary outcome, defined as a quantitative estimate of the incidence or prevalence of dog-bite injury in the pediatric population; studies reporting only secondary outcomes, without a primary outcome estimate, will be excluded. Studies enrolling both pediatric and adult participants will be included only if pediatric-specific data (aged ≤18 y) are reported separately or can be reliably extracted; otherwise, such studies will be excluded.

**Figure 2.**
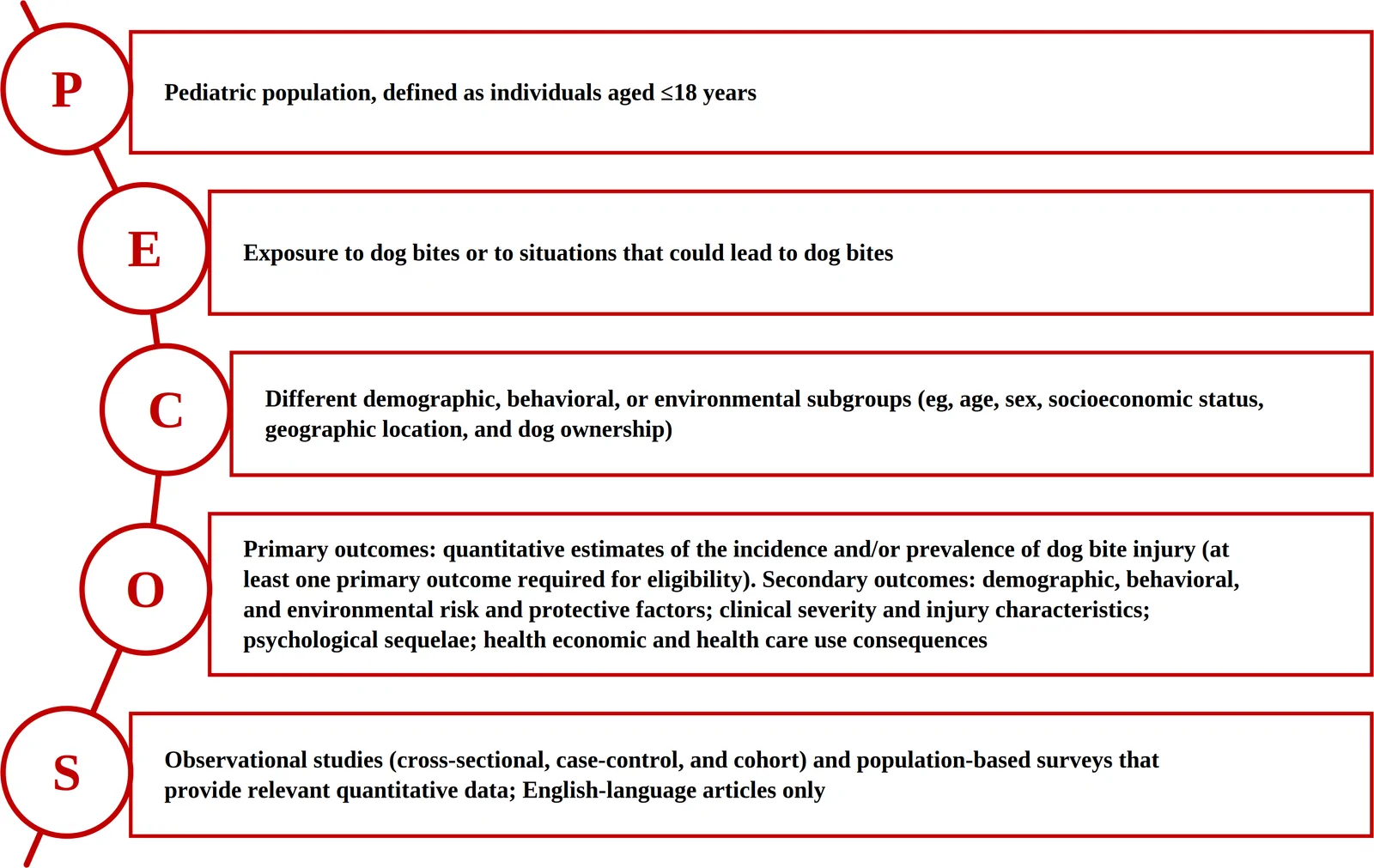
PECOS (population, exposure, comparator, outcome, and study design) eligibility framework for the systematic review. PECOS criteria govern the inclusion and exclusion of studies in a systematic review of the global incidence, prevalence, and risk factors of dog-bite injury in pediatric populations aged 18 years or younger.

Study eligibility criteria per the PECOS framework are as follows (Figure 2):

- Population: pediatric population, defined as individuals aged 18 years or younger.
- Exposure: dog bites or situations that could lead to dog bites.
- Comparator: different demographic, behavioral, or environmental subgroups (eg, age, sex, socioeconomic status, geographic location, or dog ownership).
- Outcomes: primary outcomes are quantitative estimates of the incidence and/or prevalence of dog-bite injury. Secondary outcomes are demographic, behavioral, and environmental risk and protective factors; clinical severity and injury characteristics; psychological sequelae; and health economic or health care use consequences. At least one primary outcome must be reported for a study to be eligible.
- Study design: observational studies (cross-sectional, case-control, and cohort) and population-based surveys that provide relevant quantitative data.

### Information Sources and Search Strategy

A systematic search of bibliographic databases was designed by the study investigators in consultation with a health sciences librarian and peer-reviewed against the PRESS (Peer Review of Electronic Search Strategies) criteria. The primary search was executed on August 1, 2025 in PubMed (National Library of Medicine platform), Embase (Elsevier platform), and Web of Science Core Collection (Clarivate platform), with no date restrictions applied.

Because the review is global in scope and because dog-bite epidemiology from low-income and middle-income countries is incompletely indexed in those 3 databases, the search is being extended to Scopus (Elsevier), CINAHL Complete (EBSCOhost), and the World Health Organization Global Index Medicus, which aggregates the regional indexes LILACS (Latin America and the Caribbean), African Index Medicus, the Index Medicus for the Eastern Mediterranean Region, and the Index Medicus for the South-East Asian Region. Gray literature will be sought through ProQuest Dissertations and Theses Global, the first 200 records of a Google Scholar search sorted by relevance, and the surveillance publications of national injury and public health agencies. In addition, backward citation tracking will be performed by hand-searching the reference lists of all included studies and prior systematic reviews summarized in Table 1, and forward citation tracking will be performed in Scopus and Web of Science.

Search terms combine controlled vocabulary with free-text keywords across three concept blocks: (1) dog bites and dog-related injuries; (2) pediatric populations; and (3) incidence, prevalence, and risk factors. Controlled vocabulary was mapped separately for each database using MeSH in PubMed, Emtree terms in Embase, and CINAHL subject headings in CINAHL. Terms within each concept block are combined with the Boolean operator OR, and the 3 blocks are combined with AND. The complete line-by-line search strategy for every database, together with the platform, the interface used, the date the search was executed, and the number of records retrieved, is reported in Multimedia Appendix 1 so that the search can be reproduced exactly.

Eligibility is restricted to studies published in English. This restriction was applied a priori on grounds of feasibility, as the review team does not have access to the professional translation resources required for accurate full-text data extraction from other languages, and because inaccurate extraction of numerators, denominators, and case definitions would introduce measurement error into pooled estimates. We recognize that this restriction may underrepresent evidence from regions in which dog-bite research is published predominantly in local languages, and that it is therefore a potential source of language bias that is likely to act in the direction of underrepresenting low-income and middle-income countries. To allow the magnitude and direction of this bias to be assessed rather than merely acknowledged, we will document every record that is excluded at full-text screening solely because of language, together with its country of origin and, where the abstract permits, its reported estimate, and we will report these records and their geographic distribution in the final review.

Because screening and data extraction are ongoing, the search will be re-executed in all databases immediately before the final analysis is undertaken, and any newly eligible studies identified by the updated search will be screened, extracted, appraised, and incorporated into the synthesis using the same methods. The date of the updated search and the number of additional records it yields will be reported in the final review and shown in the PRISMA 2020 flow diagram.

### Screening

All records will be imported into Rayyan, an intelligent systematic review screening tool, for deduplication and screening. Following deduplication, all records will be assessed by 2 independent reviewers against the predefined inclusion and exclusion criteria (Figure 2). Titles and abstracts will be screened first, followed by full-text review of potentially eligible articles. All reasons for exclusion will be documented at each step. A PRISMA 2020 flow diagram will be used to describe the study selection process (Figure 1).

### Data Extraction and Outcomes

Two independent reviewers will extract relevant data elements from eligible full-text articles using a standardized codebook. A double-extraction method will ensure accuracy and completeness. Discrepancies will be resolved by consensus or by a third reviewer acting as a tiebreaker. Corresponding authors of included studies will be contacted if additional data are required. The following data elements will be extracted from included studies: (1) study title; (2) study author(s); (3) publication year; (4) evidence level; (5) study design; (6) sample size; (7) sex; (8) age; (9) geographic location; (10) dog breed; (11) bite description; (12) incidence and prevalence estimates, with their numerators, denominators, and case definitions; (13) contributing and protective factors, together with the effect measure reported and the covariates used for adjustment; (14) clinical severity and injury characteristics, including anatomical site, hospital admission, operative intervention, infection, and mortality; (15) psychological sequelae and the instruments used to ascertain them; and (16) health economic and health care–use outcomes, including emergency department attendance, length of stay, and direct treatment costs. Study quality will not be extracted from the source articles but will instead be independently assessed by the review authors according to each study’s design, as described in the *Quality, Risk of Bias, and Certainty of Evidence Assessment* section.

Outcomes were defined a priori and are applied consistently across the review question, the eligibility criteria, the data extraction form, and the analysis plan. The primary outcomes are (1) the incidence of dog-bite injury in the pediatric population, expressed as events per unit of person-time or per 100,000 population per year, and (2) the prevalence or proportion of dog-bite injury in a defined pediatric population or a defined clinical denominator. The secondary outcomes are (1) demographic, behavioral, environmental, and contextual risk and protective factors associated with pediatric dog-bite injury; (2) clinical severity and injury characteristics, including anatomical site of injury, hospital admission, operative intervention, wound infection, and mortality; (3) psychological sequelae, including PTSD, anxiety, depression, phobia, and behavioral disturbance, however ascertained; and (4) health economic and health care use consequences, including emergency department attendance, length of stay, and direct treatment costs. Secondary outcomes will be extracted only from studies that are already eligible on the basis of a primary outcome and, because their measurement is expected to be highly heterogeneous, they will be synthesized narratively and in structured tables rather than pooled, unless at least 3 studies report a secondary outcome using a comparable definition and metric.

### Quality, Risk of Bias, and Certainty of Evidence Assessment

**Figure 3.**
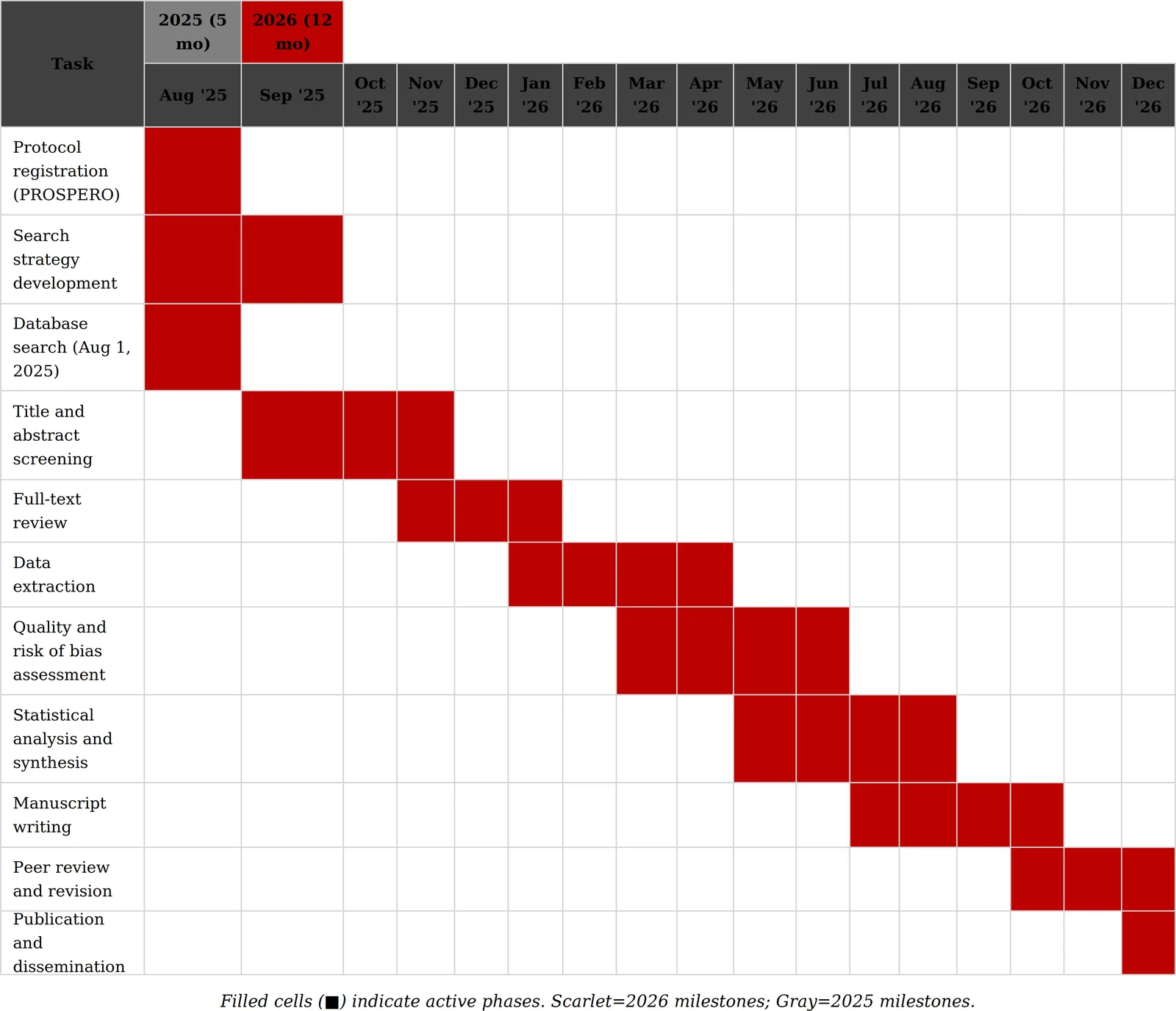
Project timeline (Gantt chart) for the systematic review. Planned phases and milestones, from PROSPERO (International Prospective Register of Systematic Reviews) registration and database searching (August 1, 2025) through screening, data extraction, quality and risk-of-bias assessment, synthesis, and dissemination, for a systematic review of the global incidence, prevalence, and risk factors for dog-bite injuries in pediatric populations (aged ≤18 y), spanning from August 2025 to December 2026.

The methodological quality and risk of bias of all included studies will be assessed using validated tools appropriate to each study design. For cross-sectional studies, the Joanna Briggs Institute (JBI) Critical Appraisal Checklist for Prevalence Studies will be applied [31]. For cohort and case-control studies, the Newcastle-Ottawa Scale will be used [32]. Quality assessment will be performed independently by 2 reviewers, with discrepancies resolved through discussion or by a third reviewer. Appraisal will be conducted at the level of individual domains rather than as a single numerical score. Every item on the relevant instrument will be rated as yes, no, unclear, or not applicable, and each study will be classified as being at low, moderate, or high overall risk of bias on the basis of the pattern of domain-level judgments, with the domains that determined the overall judgment stated explicitly. Results will be presented both as a domain-by-study matrix and as a summary table (Figure 3).

The instruments were selected to match the design of the evidence they will appraise. The JBI Critical Appraisal Checklist for Prevalence Studies is applied to cross-sectional studies and population-based surveys because it was developed specifically for questions of disease frequency and interrogates the appropriateness of the sampling frame, the sampling method, the adequacy of the sample size, the validity and standardization of case ascertainment, and the response rate, which are the features on which the validity of a prevalence estimate depends [31,33]. The Newcastle-Ottawa Scale is applied to cohort and case-control studies because it addresses the selection of the exposed and unexposed groups, the comparability of groups through design or analysis, and the ascertainment of exposure and outcome, which are the principal threats to validity in analytical observational designs [32]. Reporting practice in prevalence reviews has been shown to be inconsistent on precisely these points, which is a further reason for specifying the appraisal approach in advance [34].

Domain-level judgments will influence the synthesis in 3 prespecified ways rather than through any informal weighting. First, studies at high overall risk of bias will be excluded in sensitivity analyses, and the pooled estimates obtained with and without them will be compared directly. Second, overall risk of bias will be entered as a subgroup variable and, where at least 10 studies contribute to an analysis, as a covariate in meta-regression, so that any association between methodological quality and the estimated outcome is quantified rather than asserted. Third, the distribution of domain-level judgments will determine the risk-of-bias domain of the certainty assessment described below. Studies will not be weighted by a quality score, as quality-weighting of this kind has no established statistical basis.

The certainty of the evidence for each outcome will be assessed using the GRADE (Grading of Recommendations Assessment, Development, and Evaluation) approach [35], applying the adaptation developed for rating confidence in estimates of event rates derived from observational data [36]. For the primary outcomes of incidence and prevalence, bodies of evidence composed of observational studies will begin at high certainty because well-conducted observational studies are the appropriate design for questions of disease frequency, and will be rated down for risk of bias, inconsistency, indirectness, imprecision, and publication bias. For estimates of association between candidate risk factors and dog-bite injury, evidence will begin at low certainty, consistent with GRADE guidance for observational evidence of association, and may be rated up for a large magnitude of effect, a dose-response gradient, or if plausible residual confounding would act to reduce the observed effect. Two reviewers will make each certainty judgment independently, with disagreements resolved by discussion and, if required, by a third reviewer; the reasons for every rating decision will be documented. Certainty ratings will be presented in a GRADE summary of findings table in the final review.

### Data Analysis and Presentation

Data will be extracted using a standardized form developed prior to the analysis. Two independent reviewers will compile the extracted information, with discrepancies resolved through discussion or by a third reviewer. Findings will be presented in tabular format, organized by themes such as prevalence, incidence, injury characteristics, demographic risk factors, behavioral and environmental factors, and protective measures. Narrative synthesis will be used to identify patterns, similarities, and differences across studies. Subgroup comparisons by age group, sex, geographic region, and study design will be presented descriptively when reported by the original studies.

### Statistical Plan

#### Descriptive Analyses

Findings from all included studies will be summarized descriptively in tabular and narrative formats. Summary tables will detail study characteristics, population demographics, settings, study designs, sample sizes, outcomes, and key results. Frequencies, proportions, and ranges will be calculated to describe the prevalence and incidence of dog bites and the distribution of identified risk and protective factors. Where appropriate, means, medians, and IQRs will be reported. All analyses will be performed in R (R Foundation for Statistical Computing) using the metafor and meta packages.

#### Pooling of Primary Outcomes

Meta-analysis will be undertaken only when studies are sufficiently similar in terms of population, case definition, denominator, and outcome measurement. Pooled estimates will be derived from random-effects models fitted by restricted maximum likelihood, with the Hartung-Knapp adjustment applied to CIs, because that adjustment gives better coverage than the standard DerSimonian-Laird approach when the number of studies is small [37]. Because the variance of a raw proportion is unstable as the proportion approaches 0 or 1, study-level proportions will be transformed using the Freeman-Tukey double arcsine transformation before pooling and back-transformed to the proportion scale for presentation using the harmonic mean of the study sample sizes [38,39]; a logit transformation will be fitted as a sensitivity check, and where the 2 approaches yield materially different estimates, both will be reported. Exact (Clopper-Pearson) CIs will be calculated for individual study estimates. Incidence expressed per unit of person-time will be pooled separately from prevalence proportions, and estimates derived from population denominators will not be combined with estimates derived from facility-based denominators, since the 2 quantify different underlying parameters.

#### Pooling of Risk-Factor Estimates

Effect estimates for candidate risk and protective factors will be pooled only within a common effect measure. Odds ratios, risk ratios, and prevalence ratios will not be combined directly; where a study reports sufficient raw data, an alternative measure will be recalculated to the common metric, and where recalculation is not possible, the estimates will be synthesized in separate analyses and presented separately. Adjusted and unadjusted estimates will not be pooled together. The maximally adjusted estimate from each study will be used in the primary analysis; unadjusted estimates will be examined in a parallel sensitivity analysis; and the covariates included in each adjusted model will be tabulated so that the extent and comparability of adjustment, and therefore the scope for residual confounding, can be judged by the reader.

#### Zero Events, Multiple Estimates, and Overlapping Populations

Where the double arcsine transformation is used, proportions of 0 and 1 are accommodated without a continuity correction, and no correction will be applied. For two-by-two comparisons involving a zero cell, a continuity correction of 0.5 will be applied and the analysis repeated using exact methods as a sensitivity check; studies with 0 events in both groups contribute no information to a ratio measure and will be excluded from that analysis, while still being reported in the accompanying table. Where a single study contributes more than one eligible estimate, for example, separate estimates for several age strata or calendar periods, those estimates will not be entered into the same model as though they were independent; the most complete and representative estimate will be used in the primary analysis, and multiplicity will be addressed in secondary analyses either by combining estimates within the study before pooling or by fitting a 3-level model that accounts for the clustering of estimates within studies. Where 2 or more reports draw on the same underlying data source over an overlapping calendar period, for example, repeated analyses of a single national registry or trauma database, only one will contribute to any given pooled estimate; the report with the most complete data and the longest observation period will be retained, the decision and its rationale will be recorded in the study characteristics table, and the effect of the alternative choice will be examined in a sensitivity analysis.

#### Heterogeneity, Prediction Intervals, and When Not to Pool

Heterogeneity will be quantified using the between-study variance τ² and the *I*² statistic [40] and will be interpreted in conjunction with the pooled estimate and the observed spread of study results rather than by applying a threshold mechanically. This is particularly important for meta-analyses of prevalence, in which *I*² commonly approaches very high values even when the absolute variation between studies is modest, because the within-study variance of a proportion estimated from a large sample is very small [34]. For this reason, a 95% prediction interval will be reported alongside every pooled estimate derived from 3 or more studies, so that the range within which the true value in a comparable future population would be expected to lie is presented directly [41]. Pooling will not be undertaken when fewer than 3 studies report a comparable outcome; when case definitions, denominators, age strata, or ascertainment sources are not compatible; or when heterogeneity remains extreme and unexplained after subgroup analysis and meta-regression. In those circumstances the evidence will be presented as a structured narrative synthesis supported by summary tables and forest plots displayed without a pooled diamond, and the reason for not pooling will be stated explicitly.

#### Subgroup, Meta-Regression, and Sensitivity Analyses

A priori subgroup analyses will be conducted, where data permit, by age group (younger than 6 y, 6 to 12 y, and 13 to 18 y), sex, geographic region, country income level (high-income vs low-income and middle-income, per the World Bank classification), case definitions and ascertainment sources, study design, and overall risk of bias. When at least 10 studies contribute to an analysis, random-effects meta-regression will be used to examine these characteristics as continuous or categorical moderators, including calendar year of data collection to test for a secular trend. Sensitivity analyses will assess the robustness of pooled estimates by excluding studies at high risk of bias, excluding studies with small sample sizes or outlying estimates, comparing fixed-effects and random-effects models, comparing transformation methods, and applying the alternative selection rules described above for multiple and overlapping estimates. Small-study effects and potential publication bias will be examined using contour-enhanced funnel plots and the Egger test when at least 10 studies are available [42]; consistent with the certainty assessment, asymmetry will be interpreted as one possible explanation among several rather than as direct evidence of publication bias.

## Results

The systematic database search across PubMed, Embase, and Web of Science was conducted on August 1, 2025, with no date restrictions but limited to English-language, human studies in pediatric populations (aged ≤18 y). Screening and data extraction will be carried out in accordance with the inclusion and exclusion criteria defined in this protocol. Results will be presented as a narrative synthesis supported by descriptive statistics and summary tables, highlighting the prevalence and incidence of dog bites in children and associated risk and protective factors.

The study selection process will be summarized using a PRISMA 2020 flow diagram (Figure 1), documenting the number of records identified across PubMed, Embase, and Web of Science; duplicate records removed; records screened at the title, abstract, and full-text stages; and studies excluded with reasons, through to the final number of included studies. As screening and data extraction are ongoing at the time of submission, Figure 1 presents the structure of the study selection process, with record counts to be completed and will be updated to reflect the final counts upon completion of the review.

The anticipated completion date for data extraction and synthesis is December 2026, with dissemination planned through a peer-reviewed journal publication and presentations at relevant scientific conferences.

## Discussion

### Principal Findings and Anticipated Results

This systematic review protocol outlines a comprehensive approach to quantifying the global burden of pediatric dog bites across age subgroups and geographic regions. We anticipate that our primary findings will demonstrate that young children, particularly those under 6 years of age, bear a disproportionately high burden of dog-bite injuries globally, with injuries concentrated in the head, neck, and face. We further hypothesize that incidence and severity will vary significantly between high-income and low-income countries, reflecting differences in dog ownership practices, stray animal populations, vaccination rates, and access to trauma care. Subgroup analyses are expected to reveal distinct injury patterns and risk profiles across developmental stages, from newborns through adolescents, underscoring the need for age-targeted prevention strategies.

Existing literature on pediatric dog bites is predominantly composed of single-center retrospective studies from the United States and Western Europe, limiting both generalizability and global applicability. For instance, children under 9 years of age, particularly those under 6, consistently emerge as the most vulnerable, sustaining injuries to the head, neck, and face. Similarly, pit bull–type breeds have been reported as perpetrators in over 40% of dog bite cases, though breed attribution remains inconsistent and contested in the literature.

### Comparison With Prior Work

Compared to prior reviews, our study offers several advances. Whereas existing syntheses have been geographically constrained, our review will incorporate evidence from low-income and middle-income countries, where exposure to stray or unvaccinated dogs may substantially elevate risk. Additionally, the psychological sequelae of dog bites, including PTSD and long-term behavioral impact, have been largely underexplored in prior work, often overshadowed by immediate physical trauma. Our review explicitly captures these outcomes, addressing a meaningful gap in the current evidence base. By aggregating data across diverse settings and populations, this review moves beyond descriptive summaries to provide actionable, globally relevant insights for clinicians and policymakers.

### Implications for Clinical Practice and Public Health

The findings of this review are expected to have multifaceted implications. For clinicians, age-stratified data on injury patterns and severity will inform triage protocols and guide counseling for families following dog-bite injuries, particularly around psychological follow-up for younger children. For public health practitioners and policymakers, geographic comparisons will highlight where prevention resources are most urgently needed and where existing interventions may be contextually mismatched. Caregiver-focused education programs may be most impactful for infants and toddlers, while peer-based interventions may be more appropriate for school-age children and adolescents. The integration of psychological outcome data in this review further supports advocacy for routine mental health screening following pediatric dog-bite injuries—a practice not yet widely implemented.

### Strengths and Limitations

#### Strengths

This proposed systematic review has several important strengths. First, it will be the first comprehensive review to synthesize global evidence on pediatric dog bites, going beyond US-based or region-specific analyses. By including both high-income and low-income countries, the review will highlight geographic disparities in incidence, prevalence, and outcomes. Second, our review will employ age-stratified subgroup analyses from newborns through adolescents, allowing for identification of developmental differences in risk and injury patterns. Third, unlike many prior studies that are single-center and retrospective, this review will aggregate evidence across multiple settings, improving generalizability. Finally, by integrating findings on physical, psychological, and economic impacts, our review will provide a multidimensional perspective directly relevant to clinicians, public health practitioners, and policymakers.

#### Limitations

Several limitations should be acknowledged. First, we anticipate substantial heterogeneity across the included studies in terms of study design, populations, definitions of dog-bite severity, and outcome reporting, which may limit meaningful data pooling. Second, the review will likely be constrained by the predominance of retrospective, single-center studies, which may introduce selection bias and limit causal inference. Third, underreporting in low-income and middle-income countries, where surveillance and trauma registries are less developed, may lead to underestimation of the global burden. Fourth, inconsistencies in reporting, such as variations in injury severity scales, psychological outcomes, and breed attribution, may reduce comparability across studies. Fifth, publication bias is possible, with studies from high-income countries more likely to be published, potentially skewing the evidence base. In addition, the review was restricted to English-language publications; relevant studies published in other languages may have been missed, which could reduce the comprehensiveness and global generalizability of our findings, particularly in regions where dog-bite research is frequently published in local languages and to allow this bias to be assessed, otherwise eligible non–English records excluded at full-text screening will be counted and reported by country of origin. Finally, because several of the secondary outcomes, particularly psychological and health economic consequences, are measured inconsistently across the literature, these outcomes are expected to be synthesized narratively rather than pooled, and conclusions drawn from them will accordingly be more tentative than those drawn from the primary incidence and prevalence outcomes.

### Future Directions

This review is designed not only to synthesize existing evidence but also to catalyze future research. Key priorities identified include the need for prospective, multicenter studies in low-income and middle-income countries; standardized reporting frameworks for dog-bite severity, breed attribution, and psychological outcomes; and longitudinal studies examining the long-term mental health trajectories of pediatric dog-bite survivors. Future research should also evaluate the effectiveness of specific prevention interventions, such as dog-bite safety education programs and breed-specific legislation, across different geographic and cultural contexts.

### Dissemination Plan

Findings will be disseminated through publication in a peer-reviewed journal, with open-access publication prioritized to maximize global reach and equity of access. Results will also be presented at relevant academic conferences in pediatrics, emergency medicine, and public health. To promote translation into policy and clinical practice, findings will be communicated directly to relevant public health agencies, pediatric professional societies, and stakeholders involved in dog ownership regulation and injury prevention programming.

### Conclusions

Pediatric dog bites represent a significant, preventable public health burden with consequences that extend well beyond the immediate injury—encompassing lasting psychological trauma, health care use, and family well-being. The existing evidence base, while informative, remains geographically constrained and developmentally undifferentiated, limiting the translation of findings into globally applicable prevention strategies. This systematic review is positioned to address these gaps in a meaningful way. By synthesizing global evidence across pediatric age subgroups and geographic regions, it will generate the most comprehensive picture of the burden of pediatric dog bites to date. Critically, it will move beyond description to identify where evidence is actionable, where disparities persist, and where future investment is most needed. The findings are expected to directly inform clinical practice, public health policy, and caregiver education efforts, ultimately contributing to a reduction in the global burden of pediatric dog-bite injuries.

## Supplementary material

10.2196/103027 Multimedia Appendix 1 Full electronic search strategies.

10.2196/103027 Checklist 1 PRISMA-P checklist.

## Abbreviations

GRADE: Grading of Recommendations Assessment, Development, and Evaluation
JBI: Joanna Briggs Institute
LILACS: Latin America and the Caribbean
PECOS: population, exposure, comparator, outcome, and study design
PRESS: Peer Review of Electronic Search Strategies
PRISMA: Preferred Reporting Items for Systematic Reviews and Meta-Analyses.
PRISMA-P: Preferred Reporting Items for Systematic Review and Meta-Analysis Protocols
PROSPERO: International Prospective Register of Systematic Reviews
PTSD: posttraumatic stress disorder
