# Supplementary material for: Incidence, Prevalence, and Risk Factors of Dog Bites in Pediatric Populations: Protocol for a Systematic Review
Source: JMIR Res Protoc. 2026 Aug 27;15:e103027. doi: 10.2196/103027 (PMC13520763; doi:10.2196/103027)
Supplement: Supplementary file 1 [file resprot-v15-e103027-s001.docx]

# Multimedia Appendix 1. Full Electronic Search Strategies

The strategies below reproduce the searches exactly as executed. The primary search of PubMed, Embase, and Web of Science was run on August 1, 2025; the searches of Scopus, CINAHL, and the Global Index Medicus are being executed as an extension of the original search, and all databases will be re-run immediately before the final analysis. Record counts retrieved from each database are reported in the PRISMA 2020 flow diagram (Figure 2).

PubMed (National Library of Medicine). Searched August 1, 2025.

(("Bites and Stings"[Mesh] OR "dog bite*"[tiab] OR "dog attack*"[tiab] OR "canine bite*"[tiab] OR (("Dogs"[Mesh] OR dog[tiab] OR dogs[tiab] OR canine*[tiab]) AND (bite*[tiab] OR bitten[tiab] OR attack*[tiab] OR maul*[tiab])))) AND (("Child"[Mesh] OR "Infant"[Mesh] OR "Adolescent"[Mesh] OR "Pediatrics"[Mesh] OR child*[tiab] OR infant*[tiab] OR toddler*[tiab] OR adolescen*[tiab] OR teen*[tiab] OR youth[tiab] OR pediatric*[tiab] OR paediatric*[tiab] OR schoolchild*[tiab] OR minors[tiab])) AND (("Incidence"[Mesh] OR "Prevalence"[Mesh] OR "Epidemiology"[Mesh] OR "Risk Factors"[Mesh] OR incidence[tiab] OR prevalence[tiab] OR epidemiolog*[tiab] OR frequency[tiab] OR "risk factor*"[tiab] OR predictor*[tiab] OR determinant*[tiab] OR burden[tiab]))

Embase (Elsevier). Searched August 1, 2025.

(('dog bite'/exp OR 'dog bite*':ti,ab OR 'dog attack*':ti,ab OR (('dog'/exp OR dog:ti,ab OR dogs:ti,ab OR canine*:ti,ab) AND (bite*:ti,ab OR bitten:ti,ab OR attack*:ti,ab OR maul*:ti,ab)))) AND (('child'/exp OR 'adolescent'/exp OR 'pediatrics'/exp OR child*:ti,ab OR infant*:ti,ab OR toddler*:ti,ab OR adolescen*:ti,ab OR teen*:ti,ab OR youth:ti,ab OR pediatric*:ti,ab OR paediatric*:ti,ab)) AND (('incidence'/exp OR 'prevalence'/exp OR 'epidemiology'/exp OR 'risk factor'/exp OR incidence:ti,ab OR prevalence:ti,ab OR epidemiolog*:ti,ab OR 'risk factor*':ti,ab OR predictor*:ti,ab OR determinant*:ti,ab))

Web of Science Core Collection (Clarivate). Searched August 1, 2025.

TS=("dog bite*" OR "dog attack*" OR "canine bite*" OR ((dog OR dogs OR canine*) NEAR/3 (bite* OR bitten OR attack* OR maul*))) AND TS=(child* OR infant* OR toddler* OR adolescen* OR teen* OR youth OR pediatric* OR paediatric* OR schoolchild*) AND TS=(incidence OR prevalence OR epidemiolog* OR "risk factor*" OR predictor* OR determinant* OR burden)

Scopus (Elsevier). Extension search.

TITLE-ABS-KEY("dog bite*" OR "dog attack*" OR "canine bite*" OR ((dog OR dogs OR canine*) W/3 (bite* OR bitten OR attack* OR maul*))) AND TITLE-ABS-KEY(child* OR infant* OR toddler* OR adolescen* OR teen* OR youth OR pediatric* OR paediatric*) AND TITLE-ABS-KEY(incidence OR prevalence OR epidemiolog* OR "risk factor*" OR predictor* OR determinant*)

CINAHL Complete (EBSCOhost). Extension search.

((MH "Bites and Stings+") OR TI ("dog bite*" OR "dog attack*" OR "canine bite*") OR AB ("dog bite*" OR "dog attack*" OR "canine bite*")) AND ((MH "Child+") OR (MH "Adolescence+") OR (MH "Infant+") OR TI (child* OR infant* OR adolescen* OR pediatric* OR paediatric*) OR AB (child* OR infant* OR adolescen* OR pediatric* OR paediatric*)) AND ((MH "Incidence") OR (MH "Prevalence") OR (MH "Risk Factors") OR TI (incidence OR prevalence OR epidemiolog* OR "risk factor*") OR AB (incidence OR prevalence OR epidemiolog* OR "risk factor*"))

WHO Global Index Medicus (including LILACS, African Index Medicus, IMEMR, and IMSEAR). Extension search.

(tw:("dog bite" OR "dog bites" OR "dog attack" OR "dog attacks" OR "canine bite")) AND (tw:(child OR children OR infant OR adolescent OR pediatric OR paediatric)) AND (tw:(incidence OR prevalence OR epidemiology OR "risk factors"))

Grey literature and citation tracking. ProQuest Dissertations and Theses Global was searched using the concept blocks above; the first 200 records of a Google Scholar search sorted by relevance were screened; the reference lists of all included studies and of the systematic reviews in Table 1 were hand-searched; and forward citation tracking was performed in Scopus and Web of Science.
