# Supplementary material for: Incidence, Prevalence, and Risk Factors of Dog Bites in Pediatric Populations: Protocol for a Systematic Review
Source: JMIR Res Protoc. 2026 Aug 27;15:e103027. doi: 10.2196/103027 (PMC13520763; doi:10.2196/103027)
Supplement: Supplementary file 2 [file resprot-v15-e103027-s002.docx]

**PRISMA-P 2015 Checklist**

**PAWS: Prevalence and Warning Signs of Dog Bites -A Protocol for Systematic Review**

*Manuscript #103027 | PROSPERO Registration: CRD420251129292*

Preferred Reporting Items for Systematic review and Meta-Analysis Protocols (PRISMA-P) 2015 checklist: recommended items to address in a systematic review protocol.

| **Section and topic** | **Item No** | **Checklist item** | **Page number(s) / location in manuscript** |
| --- | --- | --- | --- |
| **ADMINISTRATIVE INFORMATION** | | | |
| Title: Identification | 1a | Identify the report as a protocol of a systematic review | Page 1 (Title). Identified as “A Protocol for Systematic Review.” |
| Title: Update | 1b | If the protocol is for an update of a previous systematic review, identify as such | Not applicable -this is a new review, not an update of a previously completed review. |
| Registration | 2 | If registered, provide the name of the registry (such as PROSPERO) and registration number | Page 1 (Trial Registration); Page 5 (Ethical Considerations; Protocol Reporting, Registration, and Amendments). PROSPERO CRD420251129292. |
| Authors: Contact | 3a | Provide name, institutional affiliation, e-mail address of all protocol authors; provide physical mailing address of corresponding author | Page 1 (author list, institutional affiliations, and corresponding author name, degrees, and e-mail). |
| Authors: Contributions | 3b | Describe contributions of protocol authors and identify the guarantor of the review | Not currently reported -see Note 2. |
| Amendments | 4 | If the protocol represents an amendment of a previously completed or published protocol, identify as such and list changes; otherwise, state plan for documenting important protocol amendments | Page 5 (Protocol Reporting, Registration, and Amendments). Two differences from the registered PROSPERO record are listed, and the plan for documenting and dating further amendments is stated. |
| Support: Sources | 5a | Indicate sources of financial or other support for the review | Page 16 (Funding). No specific grant from any public, commercial, or not-for-profit funding agency. |
| Support: Sponsor | 5b | Provide name for the review funder and/or sponsor | Page 16 (Funding). Not applicable -no funder or sponsor. |
| Support: Role of sponsor or funder | 5c | Describe roles of funder(s), sponsor(s), and/or institution(s), if any, in developing the protocol | Page 16 (Funding). Not applicable -the review is unfunded. |
| **INTRODUCTION** | | | |
| Rationale | 6 | Describe the rationale for the review in the context of what is already known | Pages 2–4 (Introduction), including the gap analysis of existing systematic reviews in Table 1 (Page 4). |
| Objectives | 7 | Provide an explicit statement of the question(s) the review will address with reference to participants, interventions, comparators, and outcomes (PICO) | Page 1 (Abstract -Objectives); Page 5 (Review Question); Page 6 (Inclusion and Exclusion Criteria; Figure 1). See Note 3 regarding PECOS. |
| **METHODS** | | | |
| Eligibility criteria | 8 | Specify the study characteristics (such as PICO, study design, setting, time frame) and report characteristics (such as years considered, language, publication status) to be used as criteria for eligibility for the review | Pages 5–6 (Inclusion and Exclusion Criteria) and Figure 1 (Page 6); Page 7 for date and English-language restrictions. |
| Information sources | 9 | Describe all intended information sources (such as electronic databases, contact with study authors, trial registers or other grey literature sources) with planned dates of coverage | Pages 6–7 (Information Sources and Search Strategy) -six databases with named platforms, WHO Global Index Medicus regional indexes, grey literature, citation tracking, and author contact; search executed August 1, 2025, with no date restrictions and re-executed before final analysis. |
| Search strategy | 10 | Present draft of search strategy to be used for at least one electronic database, including planned limits, such that it could be repeated | Page 7 (Information Sources and Search Strategy); complete line-by-line strategies for every database in Multimedia Appendix 2. Peer reviewed against PRESS criteria (Page 6). |
| Study records: Data management | 11a | Describe the mechanism(s) that will be used to manage records and data throughout the review | Page 7 (Screening) -Rayyan for deduplication and screening; standardized codebook and extraction form (Pages 7–8; Page 9). |
| Study records: Selection process | 11b | State the process that will be used for selecting studies (such as two independent reviewers) through each phase of the review (that is, screening, eligibility and inclusion in meta-analysis) | Page 7 (Screening) -two independent reviewers at title/abstract and full-text stages, exclusions documented, summarized in Figure 2 (Page 12). |
| Study records: Data collection process | 11c | Describe planned method of extracting data from reports (such as piloting forms, done independently, in duplicate), any processes for obtaining and confirming data from investigators | Pages 7–8 (Data Extraction and Outcomes) -duplicate independent extraction, third-reviewer tiebreak, and contact with corresponding authors; see also Page 9. |
| Data items | 12 | List and define all variables for which data will be sought (such as PICO items, funding sources), any pre-planned data assumptions and simplifications | Pages 7–8 (Data Extraction and Outcomes)- sixteen defined data elements. Study quality is not extracted but independently appraised (Page 8). |
| Outcomes and prioritization | 13 | List and define all outcomes for which data will be sought, including prioritization of main and additional outcomes, with rationale | Page 8 (Data Extraction and Outcomes) -two primary and four secondary outcomes defined a priori with prioritization rationale; see also Page 6 and Page 1. |
| Risk of bias in individual studies | 14 | Describe anticipated methods for assessing risk of bias of individual studies, including whether this will be done at the outcome or study level, or both; state how this information will be used in data synthesis | Pages 8–9 (Quality, Risk of Bias, and Certainty of Evidence Assessment) -domain-level appraisal using JBI (prevalence) and Newcastle-Ottawa (cohort/case-control), with the three prespecified routes by which judgments influence synthesis (Page 9). |
| Data synthesis | 15a | Describe criteria under which study data will be quantitatively synthesised | Page 10 (Statistical Plan -Pooling of primary outcomes); Page 11 for conditions under which pooling will not be undertaken. |
| Data synthesis | 15b | If data are appropriate for quantitative synthesis, describe planned summary measures, methods of handling data and methods of combining data from studies, including any planned exploration of consistency (such as I2, Kendall’s τ) | Pages 10–11 (Statistical Plan) -random-effects REML with Hartung-Knapp, Freeman-Tukey double arcsine transformation, handling of zero events, multiple and overlapping estimates, and adjusted versus unadjusted estimates; consistency explored with τ² and I² with prediction intervals. Software: R, metafor and meta packages (Page 9). |
| Data synthesis | 15c | Describe any proposed additional analyses (such as sensitivity or subgroup analyses, meta-regression) | Page 11 (Subgroup, meta-regression, and sensitivity analyses). |
| Data synthesis | 15d | If quantitative synthesis is not appropriate, describe the type of summary planned | Page 11 (when not to pool) -structured narrative synthesis with summary tables and forest plots without a pooled diamond; see also Pages 8 and 9. |
| Meta-bias(es) | 16 | Specify any planned assessment of meta-bias(es) (such as publication bias across studies, selective reporting within studies) | Page 11 (Statistical Plan) -contour-enhanced funnel plots and the Egger test where at least ten studies are available; language bias quantified per Page 7. |
| Confidence in cumulative evidence | 17 | Describe how the strength of the body of evidence will be assessed (such as GRADE) | Page 9 (Quality, Risk of Bias, and Certainty of Evidence Assessment) -GRADE, using the adaptation for event rates from observational data, with a Summary of Findings table. |

**Notes**

1. Page numbers refer to the clean revised manuscript submitted in this round. Page numbers should be reconfirmed against the final submitted file, as pagination can shift with formatting.

2. Item 3b is not currently addressed in the manuscript. PRISMA-P requires a description of each author’s contribution to the protocol and explicit identification of the guarantor of the review. An Authors’ Contributions section should be added before submission and this row updated with its page number.

3. Item 7 refers to PICO. This review synthesizes observational evidence on disease frequency and accordingly uses the Population, Exposure, Comparator, Outcome, and Study Design (PECOS) framework, which serves the equivalent function.

*The copyright for PRISMA-P (including the checklist) is held by the PRISMA-P Group and is distributed under a Creative Commons Attribution Licence 4.0.*

*From: Shamseer L, Moher D, Clarke M, Ghersi D, Liberati A, Petticrew M, Shekelle P, Stewart L, PRISMA-P Group. Preferred reporting items for systematic review and meta-analysis protocols (PRISMA-P) 2015: elaboration and explanation. BMJ. 2015 Jan 2;349(jan02 1):g7647.*
